# Supplementary material for: Meiosis I progression in spermatogenesis requires a type of testis-specific 20S core proteasome
Source: Nat Commun. 2019 Jul 29;10:3387. doi: 10.1038/s41467-019-11346-y (PMC6662770; doi:10.1038/s41467-019-11346-y)
Supplement: Supplementary file 1 — Supplementary Information [file 41467_2019_11346_MOESM1_ESM.pdf]

SUPPLEMENTARY INFORMATION

Meiosis I Progression in Spermatogenesis Requires a Type of Testis-specific 20S Core Proteasome

Qianting Zhang[1], Shu-Yan Ji[2], Kiran Busayavalasa[1], Jingchen Shao[1], Chao Yu[1,#]

SUPPLEMENTARY FIGURES

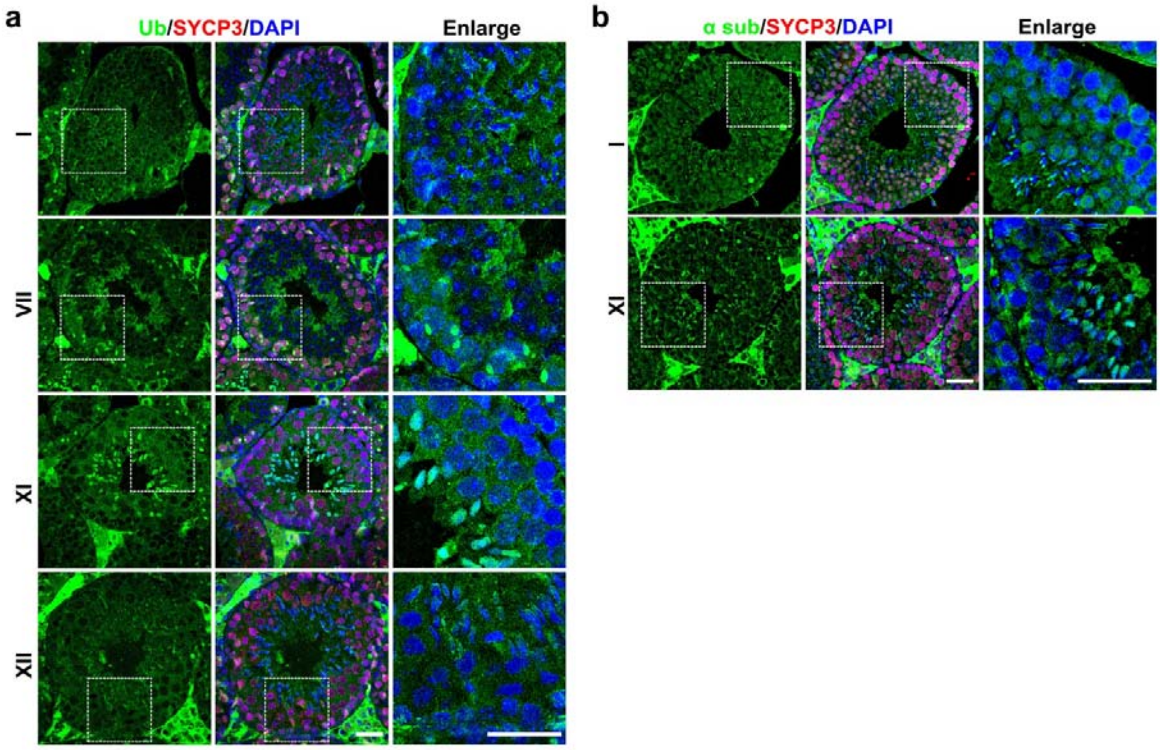

Supplementary Fig. 1. Dynamics of protein ubiquitination (Ub) and α subunits of proteasomes during spermatogenesis. a–b, Immunofluorescent staining of Ub (**a**) and α subunits of 20S core proteasome (α sub, **b**) in sections of PD42 testes. Different stages of seminiferous tubules are shown. The regions bordered with dashed lines are enlarged in the right. At least three testes were stained and analyzed. Scale bars, 25 µm.

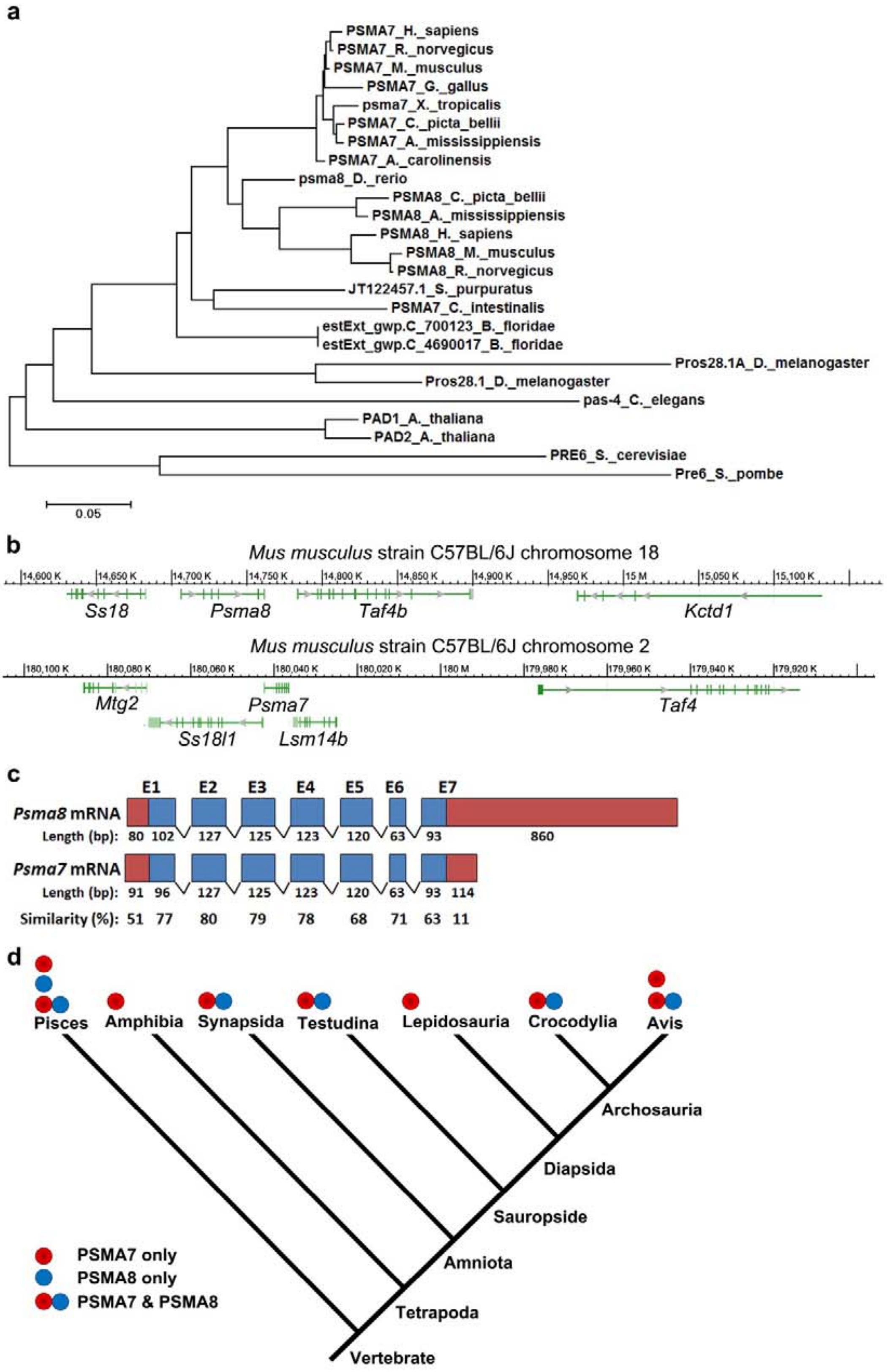

a
PSMA7_H._sapiens
PSMA7_R._norvegicus
PSMA7_M._musculus
PSMA7_G._gallus
psma7_X._tropicalis
PSMA7_C._picta_bellii
PSMA7_A._mississippiensis
PSMA7_A._carolinensis
psma8_D._rerio
PSMA8_C._picta_bellii
PSMA8_A._mississippiensis
PSMA8_H._sapiens
PSMA8_M._musculus
PSMA8_R._norvegicus
JT122457.1_S._purpuratus
PSMA7_C._intestinalis
estExt_gwp.C_700123_B._floridae
estExt_gwp.C_4690017_B._floridae
Pros28.1A_D._melanogaster
Pros28.1_D._melanogaster
pas-4_C._elegans
PAD1_A._thaliana
PAD2_A._thaliana
PRE6_S._cerevisiae
Pre6_S._pombe
0.05
b
Mus musculus strain C57BL/6J chromosome 18
Ss18
Psma8
Taf4b
Kctd1
Mus musculus strain C57BL/6J chromosome 2
Mtg2
Psma7
Taf4
Ss18l1
Lsm14b
c
E1 E2 E3 E4 E5 E6 E7
Psma8 mRNA
Length (bp): 80 102 127 125 123 120 63 93 860
Psma7 mRNA
Length (bp): 91 96 127 125 123 120 63 93 114
Similarity (%): 51 77 80 79 78 68 71 63 11
d
Pisces
Amphibia
Synapsida
Testudina
Lepidosauria
Crocodylia
Avis
Archosauria
Diapsida
Sauropside
Amniota
Tetrapoda
Vertebrate
PSMA7 only
PSMA8 only
PSMA7 & PSMA8

Supplementary Fig. 2. Evolutionary scenery of *Psma7* and *Psma8*. a, Phylogenetic tree showing the evolutional relevance of PSMA7 and PSMA8 from different species. The protein sequences were downloaded from NCBI. The evolutionary history was inferred using the Neighbor-Joining method. Scale bar, 0.05 million years. **b–c,** Schematic diagram showing the genome fragments containing *Psma7* and *Psma8* **(b),** and the exon structures of *Psma7* and *Psma8* **(c)** in mouse. **d,** Schematic diagram showing the presence of PSMA7 and PSMA8 during evolution.

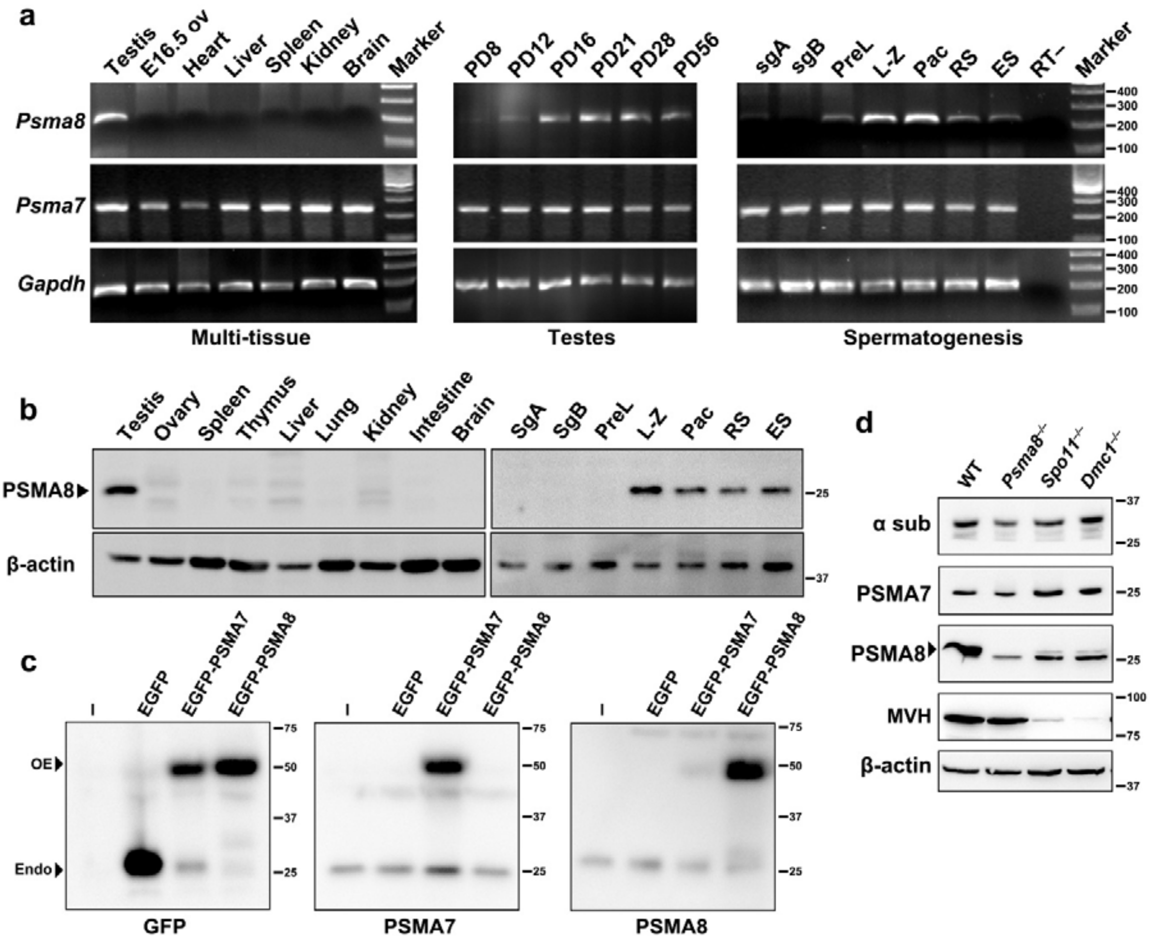

Supplementary Fig. 3. Expression of PSMA8 in mouse. a–b, Semi-quantitative PCR (**a**) and Western blotting (**b**) results showing specific expression of *Psma8* and *Psma7* in spermatocytes entering meiosis in testes. E16.5 ov, ovaries at embryonic day 16.5; PD, postnatal day; sgA, spermatogonia A; sgB, spermatogonia B; PreL, pre-leptonema; L-Z, leptonema-zygonema; Pac, pachynema; RS, round spermatids; ES, elongated spermatids. **c,** Western blotting detected the endogenous and overexpressed PSMA7 and PSMA8. OE, over-expression; Endo, endogenous. **d,** Western blotting showing the expression of PSMA8 in *Spo11*$^{-/-}$ and *Dmc1*$^{-/-}$ testes at PD21. Arrowhead indicates the PSMA8 band.

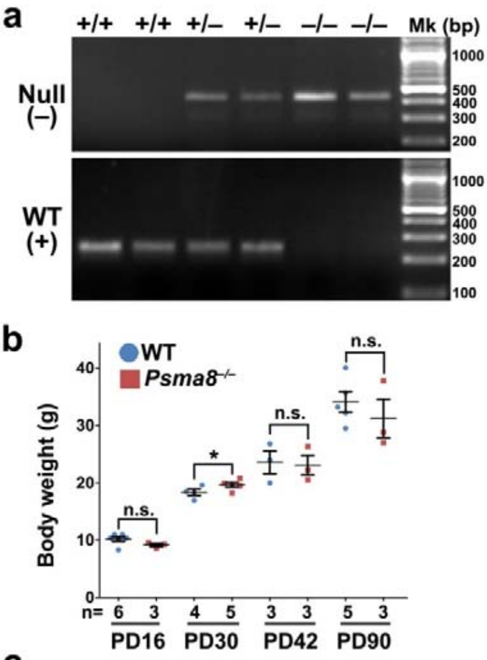

c

Breeding	Pups/litter	Litters
♂+/– X ♀+/–	7.57 (±0.36)	45
♂+/– X ♀–/–	7.57 (±0.50)	30
♂–/– X ♀+/–	0*	0

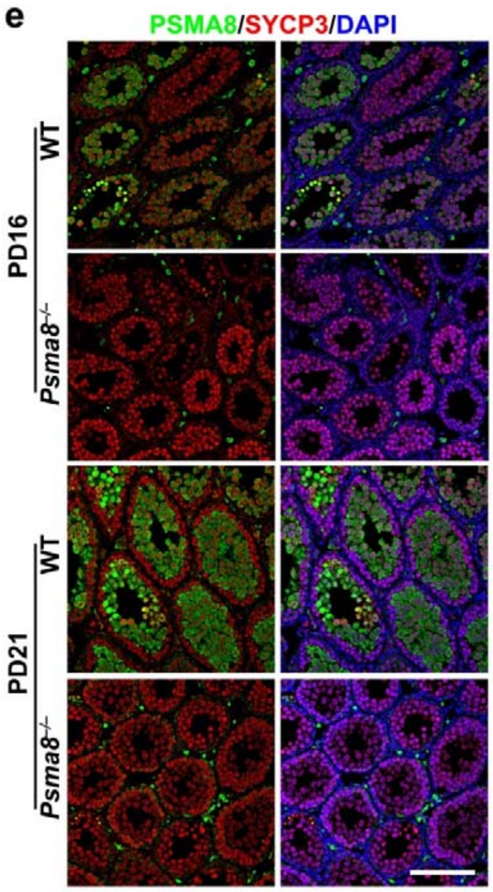

d

Breeding	+/+	+/–	–/–	total
♂+/– X ♀+/–	54 (25.1%)	114 (53.0%)	47 (21.9%)	215
♂+/– X ♀–/–	0	73 (52.5%)	66 (47.5%)	139

Supplementary Fig. 4. Generation of *Psma8* knockout mice. a, Genotyping result for the WT (+) and null (–) alleles of *Psma8*. +/+, +/– and –/– represent Wild-type (WT), heterozygous and homozygous mice, respectively. Markers (Mk) of DNA were indicated. **b,** Body weights of WT and *Psma8*$^{-/-}$ mice at indicated ages. The numbers of mice analyzed (n) were indicated. Error bar indicated S.E.M. * P<0.001 by two-tailed Student's *t* tests. n.s., not significant. **c,** Pups/litter and litter numbers analyzed for indicated breeding. *, no pups or pregnancies were observed through a 3-month breeding. n > 6 for each breeding set. **d,** Numbers and percentages of *Psma8*$^{+/+}$, *Psma8*$^{+/-}$ and *Psma8*$^{-/-}$ pubs from *Psma8*$^{+/-}$ male to *Psma8*$^{+/-}$ female breeding, or *Psma8*$^{+/-}$ male to *Psma8*$^{-/-}$ female breeding. **e**, Immunofluorescent staining showing successful deletion of PSMA8 in spermatocytes at PD16 and PD21. Scale bar, 100 μm.

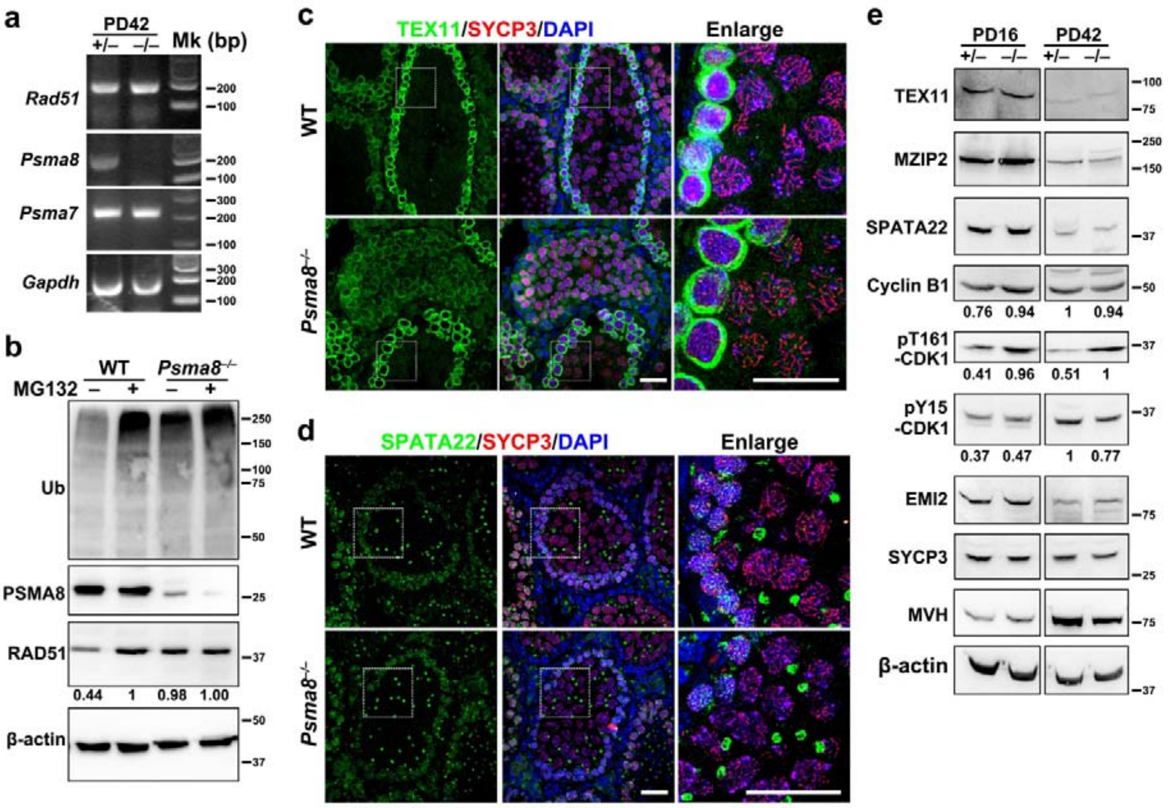

Supplementary Fig. 5. Effects of PSMA8 deletion on protein ubiquitination and protein degradation. a, Real-time PCR results showing the levels of indicated genes in WT and *Psma8*$^{-/-}$ testes at PD42. **b,** Western blotting showing the effects of MG132 on WT and *Psma8*$^{-/-}$ testes. Spermatocytes were treated with 50 µM MG132 for 4 h before sample preparation for Western blotting. RAD51 band intensity is quantified from three different blottings and the average is shown underneath the blotting. **c–d,** Immunofluorescent staining of TEX11 (**c**) and SPATA22 (**d**) in testes sections derived from WT and *Psma8*$^{-/-}$ males at PD25. Scale bars, 50 µm. **e,** Western blotting results showing the levels of indicated proteins in WT and *Psma8*$^{-/-}$ testes at the ages of PD16 and PD42. Quantification of the intensity of Western blotting bands is shown under each blotting.

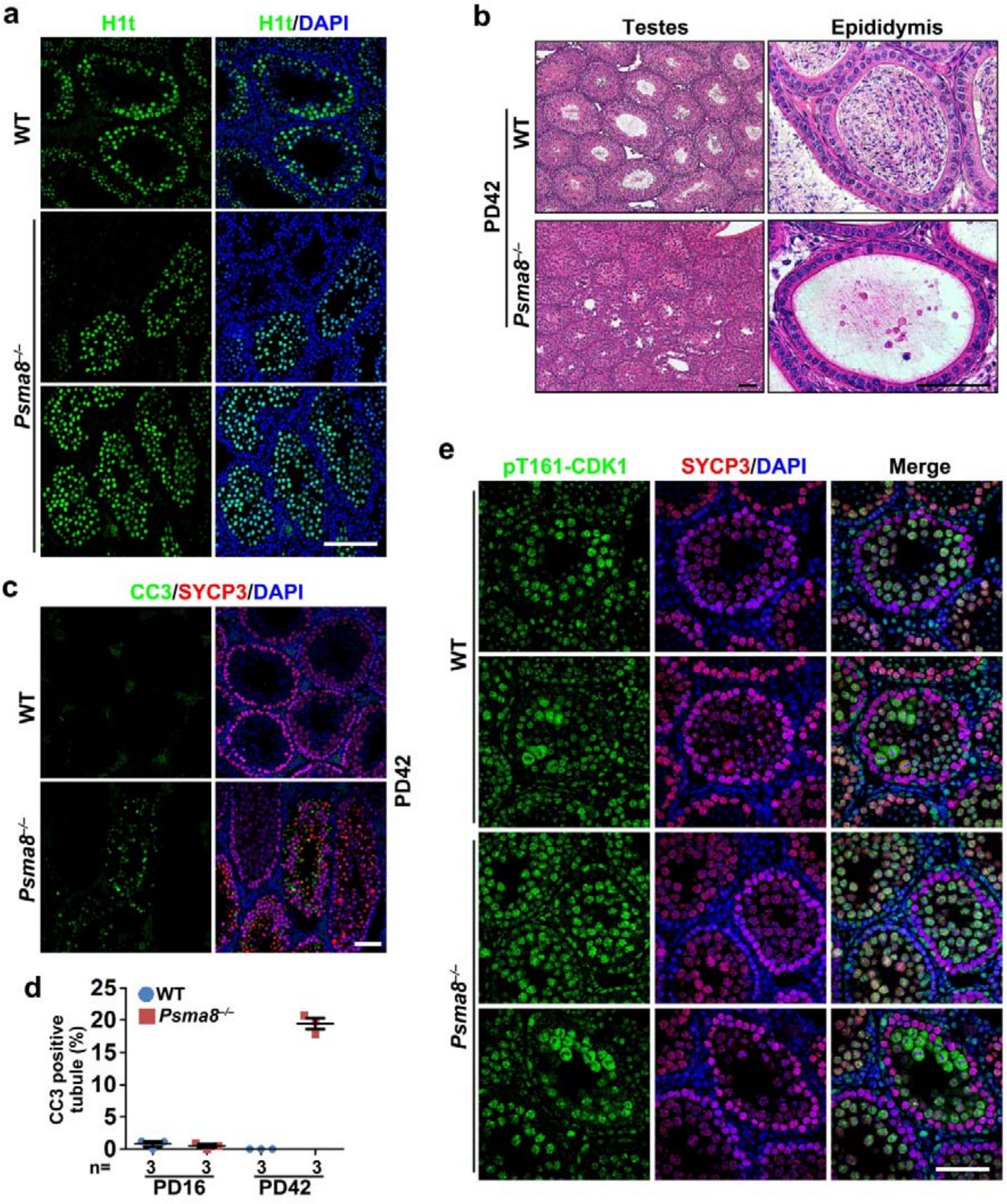

Supplementary Fig. 6. Meiotic prophase I was less affected by PSMA8 deletion. a, Staining of H1t showing that PSMA8-null spermatocytes progressed into late-pachytene stage. Scale bar, 50 µm. **b,** Hematoxylin & eosin (H&E) staining of testes and epididymis derived from WT and *Psma8*$^{-/-}$ males. Magnified images of testes were shown in Figure 6a. Scale bars, 50 µm. **c–d,**

Staining of cleaved caspase 3 (CC3) showing the massive apoptosis after PSMA8 deletion (**c**), and the quantification of tubules containing massive CC3 signal was shown in (**d**). Scale bar, 50 μm. Error bars indicated S.E.M. The numbers (n) of sections quantified are indicated. **e,** Staining of CDK1 phosphorylated at threonine 161 (pT161-CDK1) showing activation of CDK1 in indicated testes sections at PD25. Scale bar, 50 μm.

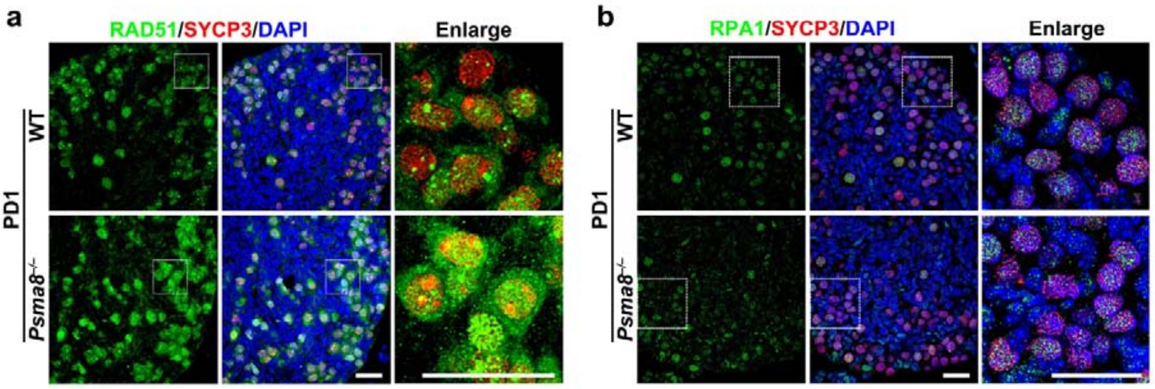

Supplementary Fig. 7. RAD51 and RPA1 remained stable in WT and *Psma8*$^{-/-}$ oocytes. a–b, Immunostaining of RAD51 (**a**) and RPA1 (**b**) on WT and *Psma8*$^{-/-}$ ovary sections at PD1. Scale bars, 25 μm.

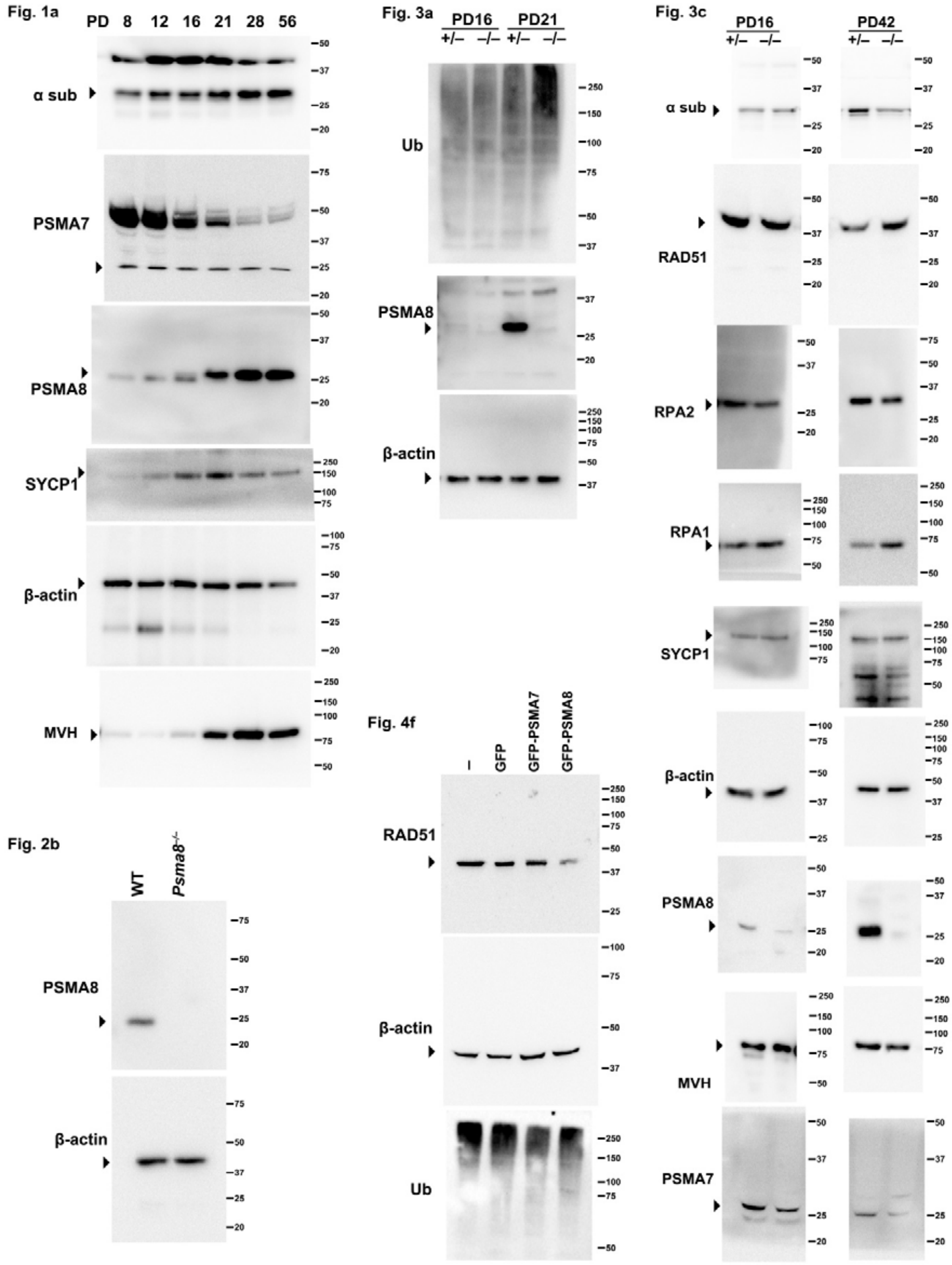

Supplementary Fig. 8. Uncropped images of Western blotting results.

Supplementary Table 1. Primer sequences.

Primer name	Genes targeted	Application	Sequences (5'-3')
P1	*Psma8*	Genotyping (443bp for WT)	5'-AAGGTTCTCTTTGTCATACTTGTCC-3'
P2			5'- CCTTTTAAACATAGATGACCCTTTG-3'
P3	*Psma8*	Genotyping (with P1; 200bp for WT allele)	5'-TTTTTTCTACCCCAAGCACAA-3'
P4	*Psma8*	Genotyping (400bp for null allele)	5'-ATTCGAGGAACTAATATAGCTTGG-3'
P5			5'-ATGCAAAAACCCACACATGTATAC-3'
S522	*Dmc1*	Genotyping (233bp/147bp for WT/null allele)	5'-CCGGCCAGATTACATTTCTT-3'
S523			5'-AAAGGGACTGCTGAGGCATA-3'
S524			5'-GCCAGAGGCCACTTGTGTAG-3'
S519	*Spo11*	Genotyping (165bp/200bp for WT/null allele)	5'-CTGCTCAGGGAGGAGAACAC-3'
S520			5'-TCAGGACAGGGCATAGCAGT-3'
S521			5'-GCCAGAGGCCACTTGTGTAG-3'
S185	*Psma8*	RT-PCR (215bp)	5'-CGAGGAACTAATATAGTTGTGCTTG-3'
S186			5'-ATATACTCTACAGTGACGGGATCCT-3'
C017	*Psma7*	RT-PCR (217bp)	5'-CCAAGTCAGTGCGTGAATTTC-3'
C018			5'-TCTTTCTCCTTCTCAATTTCAGC-3'
C051	*Rad51*	RT-PCR (186bp)	5'-TTTGGTGTCGCAGTGGTAATC-3'
C052			5'-AAGACAGGGAGAGTCATAGATTTTG-3'
Z531	*Gapdh*	RT-PCR (181bp)	5'-ACACTGAGGACCAGGTTGTCTC-3'
Z532			5'-TACTCCTTGGAGGCCATGTAG-3'
