# Supplementary material for: Meiosis I progression in spermatogenesis requires a type of testis-specific 20S core proteasome
Source: Nat Commun. 2019 Jul 29;10:3387. doi: 10.1038/s41467-019-11346-y (PMC6662770; doi:10.1038/s41467-019-11346-y)
Supplement: Supplementary file 3 — Description of Additional Supplementary Files [file 41467_2019_11346_MOESM3_ESM.pdf]

Description of Additional Supplementary Files

File Name: Supplementary Data 1
Description: The information of antibodies used.
